# All deaths follow recurrence, but not all recurrences are fatal: a retrospective cohort study of recurrence timing and survival after curative resection of hepatocellular carcinoma

**DOI:** 10.3389/fonc.2026.1991522

**Published:** 2026-10-28

**Authors:** Qian Zhang, Ziyu Hou, Jie Zhang, Lunan Qi, Dawei Deng, Bangde Xiang

**Affiliations:** 1 Department of Hepatobiliary Surgery, North Sichuan Medical College Affiliated Hospital, Nanchong, China; 2 Department of Hepatobiliary Surgery, Guangxi Medical University Cancer Hospital, Nanning, Guangxi, China

**Keywords:** circulating tumour cells, epithelial-mesenchymal transition, hepatocellular carcinoma, microvascular invasion, overall survival, recurrence, surveillance

## Abstract

**Background:**

Postoperative recurrence is the main barrier to long-term survival after curative resection of hepatocellular carcinoma (HCC). Whether recurrence timing, rather than recurrence itself, distinguishes lethal from non-lethal trajectories remains unclear.

**Methods:**

We analyzed 154 patients after curative HCC resection at a single centre. Preoperative circulating tumour cells (CTCs) were classified as epithelial, mixed, or mesenchymal (M-CTC); microvascular invasion (MVI) was graded M0-M2. We defined the dissemination axis (DA) as a composite of the preoperative M-CTC phenotype and postoperative pathological MVI grade. Because MVI is pathological, the complete DA can only be calculated after pathologic review, not before surgery. Early recurrence was defined as recurrence within 24 months. Overall survival (OS) and recurrence-free survival (RFS) were assessed with penalised Cox models, landmark and time-window analyses, competing-risk methods, and exploratory log-hazard mediation.

**Results:**

Of 154 patients, 142 (92.2%) developed recurrence: 118 with early recurrence, 24 with late recurrence, and 12 remained recurrence-free. During the observed follow-up period, no deaths occurred without prior recurrence (0/154); 80 of 83 deaths (96.4%) were preceded by early recurrence. Median OS was 18.5 months for early recurrence and was not reached for late or non-recurrence groups (observed median survival, 49.0 and 39.5 months respectively). Across DA strata, 24-month recurrence rates increased from 22.2% (low risk) to 95-98% (high risk), while median OS decreased from 47 to 12 months. High DA status independently predicted poorer RFS (HR 3.72; 95% CI 2.28-6.07) and OS (HR 3.25; 95% CI 1.69-6.25). The effect was concentrated in the first 24 months. Early recurrence accounted for 27.5% of the log-hazard attenuation (95% CI 15.5-44.9%), but this exploratory analysis cannot establish causal mediation. 24-month RFS status discriminated subsequent OS (time-dependent AUCs 0.732-0.862).

**Conclusion:**

In this exploratory single-centre retrospective cohort study, recurrence timing distinguished lethal from non-lethal outcomes. The DA, combining a preoperative CTC phenotype with the postoperative MVI grade, identified patients at high risk of 24-month early recurrence, supporting evaluation of risk-stratified surveillance and 24-month RFS as an early endpoint in perioperative HCC trials. Because no external validation was performed, these findings are hypothesis-generating and require independent validation before clinical use.

## Introduction

Hepatocellular carcinoma (HCC) is one of the leading global causes of cancer-related death (1). Surgical resection offers the highest curative potential for early- or intermediate-stage HCC (2). Post-resection recurrence remains a key clinical challenge: nearly 75% of recurrences occur within two years post-surgery, and these early recurrences have the poorest prognosis (3, 4). Therefore, clinical management needs accurate stratification of both the likelihood and the expected timing of patient recurrence.

Early and late recurrences are not the same disease event. Early recurrence is thought to reflect occult micrometastasis and vascular dissemination present at the time of surgery; late recurrence more often represents *de novo* tumours arising in a diseased liver (5). This distinction has practical consequences. A risk-stratification test that identifies the early-recurrence trajectory could guide adjuvant treatment and the intensity of imaging in the first one to two years; because such a test cannot identify late recurrence, long-term surveillance remains a separate obligation.

Circulating tumour cells allow cell-based characterization of cancer dissemination. In HCC, circulating tumour cells (CTCs) that underwent epithelial-mesenchymal transition (EMT) are more invasive and more strongly correlated with tumour recurrence than total CTC counts (6–10). When graded via the M0/M1/M2 staging system rather than classified as a binary variable, microvascular invasion reflects this underlying biological mechanism at the pathological level (11, 12). Multiple groups have reported associations between CTC phenotypes, microvascular invasion and early recurrence (7, 12–17), and CTC phenotypes are also linked to pathological vascular features including vessels encapsulating tumour clusters (18). Recent narrative and systematic reviews have summarized the clinical utility of CTC phenotyping for HCC management (19–21). However, few existing studies include both recurrence timing and overall survival in a unified analytical framework, and no study to date has directly tested whether a dissemination axis combining a preoperative CTC phenotype with postoperative pathological MVI grade affects overall survival primarily through early recurrence. Recent studies instead focus on longitudinal CTC monitoring for minimal residual disease (22) or CTC-based composite scoring systems for recurrence risk prediction (23); neither approach investigates whether a single integrated dissemination axis (DA) can identify the recurrence-timing pattern that drives survival outcomes.

The present study differs from prior research in three core aspects. First, it conceptualizes recurrence as a time-stratified clinical classification rather than a discrete event. Second, it establishes a unified DA that integrates preoperative EMT-phenotype CTC measurements with postoperative pathological MVI grade; this integrated metric can be computed shortly after surgery without the requirement for sequential postoperative sampling (22, 23). Third, it explores the association between recurrence timing and overall survival via mediation analysis, competing-risk analysis, and surrogate-endpoint analysis. Accordingly, we investigated in a cohort of 154 patients with complete OS data whether recurrence timing distinguishes lethal from non-lethal disease trajectories and whether the DA identifies the high-risk early-recurrence pattern. This work provides hypothesis-generating evidence to support evaluation of risk-stratified postoperative surveillance and adjuvant clinical trial enrichment.

## Materials and methods

### Study design and participants

This retrospective study screened 810 patients who underwent resection for hepatocellular carcinoma at Guangxi Medical University Cancer Hospital between August 2015 and June 2019; after excluding 656 patients, the final analytic cohort comprised 154 patients. Participant flow and exclusion reasons are displayed in **Supplementary Figure S1**. The most common reason for exclusion was absence of preoperative CTC data (n=563); no additional patients were separately recorded as excluded for missing MVI data. All included patients had complete preoperative CTC enumeration, MVI grading, cytokeratin 19 (CK19) immunohistochemistry data, and overall survival (OS) follow-up records. Because the source dataset contained only the final analytic cohort, baseline characteristics of excluded patients were not available for formal comparison. The cohort partially overlaps our previously published 105-patient cohort (8). This study complied with the Declaration of Helsinki, was approved by the Institutional Ethics Committee of Guangxi Medical University Cancer Hospital (Approval No. KY2026017), and obtained written informed consent from all participants.

### CTC enumeration and phenotyping

5 mL peripheral venous blood was collected 1–2 days before surgery. CTCs were enriched via the CanPatrol nanofiltration platform, then categorized by RNA *in situ* hybridisation against epithelial (EpCAM, CK8/18/19) and mesenchymal (Vimentin, Twist) markers; CD45 staining was used to exclude leukocytes (6, 8, 24). CTCs expressing only epithelial markers were classified as epithelial CTCs (E-CTC), those with only mesenchymal markers as M-CTCs, and those with both as mixed CTCs. We adopted predefined cutoffs from our previous study: M-CTC positivity is defined as ≥1 M-CTC per 5 mL blood, and total CTC positivity as ≥6 CTCs per 5 mL blood (8, 14). These cutoffs were not re-optimized for the current cohort.

### Pathological evaluation

Two senior pathologists, blinded to clinical outcomes, independently reviewed the resection specimens. MVI was defined as microscopically visible tumour cell nests within endothelial-lined vascular spaces, with portal vein branch invasion (including capsular vessels) the most common pattern in HCC (25). MVI is stratified into three grades: M0 (no MVI detected), M1 (low-risk MVI: ≤5 MVI foci, all within 1 cm of the primary tumor in peritumoral hepatic tissue), and M2 (high-risk MVI: >5 peritumoral MVI foci without distant peritumoral tissue invasion; or MVI detected in peritumoral hepatic tissue over 1 cm from the primary tumor) (26). CK19 expression was evaluated via immunohistochemistry, and positive expression was defined as the presence of staining in >5% of tumor cells (8).

### Follow-up and outcome definitions

Patients were followed up every 1–2 months in the first post-treatment year, and every 3 months after that. Follow-up assessments included serum alpha-fetoprotein (AFP) testing, abdominal ultrasonography, and dynamic computed tomography (CT) or magnetic resonance imaging (MRI). Tumour recurrence was defined as a new intrahepatic/extrahepatic lesion with imaging features matching HCC, independent of AFP level changes. Recurrence-free interval was calculated from the date of surgery to the date of confirmed recurrence; patients who remained recurrence-free were censored at the time of their last recurrence assessment. Median RFS is an event-driven outcome estimate and should not be interpreted as the median follow-up duration. OS was calculated from surgery to death from any cause, or to the last follow-up for censored patients. Clinical follow-up duration was defined as the interval from surgery to the last clinical assessment. The causes of death were not adjudicated, and in the de-identified dataset, administrative censoring events cannot be differentiated from loss to follow-up. Early recurrence was defined as recurrence within 24 months after surgery, late recurrence as recurrence after 24 months, and recurrence-free status as no confirmed recurrence throughout follow-up. This classification is defined as the recurrence-time phenotype, which encompasses three categories: early recurrence, late recurrence, and no recurrence. It is a descriptive clinical classification based on observed recurrence timing, and does not infer an underlying molecular or biological phenotype. In a prespecified sensitivity analysis, we adjusted the late recurrence threshold to 36 months to test the robustness of our findings. Since early recurrence definitions vary widely across previous studies (some use a 12-month cutoff (27)), we prespecified a 24-month cutoff in our study protocol and tested an alternative 36-month cutoff to confirm the stability of our conclusions.

### Risk axes

A dissemination axis (DA) integrating the preoperative M-CTC phenotype and postoperative pathological MVI grade can stratify patients into low-risk, intermediate-risk, and high-risk groups. Of note, the complete DA score can only be calculated after postoperative pathological assessment, rather than preoperatively. The component thresholds were pre-specified based on prior literature and were not re-optimized in the present cohort. The three-level combination rule was defined prior to outcome modeling; however, it was not a formally registered protocol endpoint and has not undergone external validation. Accordingly, this rule is treated as an exploratory composite endpoint. The rule was formulated to reflect concordant biological evidence of vascular dissemination and was not selected by fitting alternative combinations to the recurrence or survival outcomes of this cohort. Low risk = M-CTC-negative and MVI M0; intermediate risk = only one positive component between M-CTC positivity and MVI M1; high risk = either MVI M2 or concurrent M-CTC positivity and MVI M1. Because MVI is a pathological variable, the complete DA is not available before surgery; it can only be calculated after pathologic review. The host-liver axis is an exploratory background variable defined as co-occurrence of cirrhosis and hepatitis B virus (HBV) DNA load >= 500 IU/mL. We did not hypothesize that this axis predicts early recurrence, and we report this result only to show that it cannot act as a surrogate for tumour biological characteristics.

### Statistical analysis

All analyses were pre-specified and reported per the STROBE statement; prediction model components followed TRIPOD guidelines (28), and tumour marker analyses followed REMARK guidelines (29, 30). Continuous variables were summarized as medians and compared via the Mann-Whitney U test; categorical variables were compared via the chi-square test, with Fisher’s exact test for small expected cell counts. Kaplan-Meier estimation and log-rank tests were used to characterize recurrence-free survival (RFS) and OS.

Cox proportional hazards models with ridge penalty were fitted to stabilize coefficient estimates under near-complete separation (31). The primary analysis used a penalty parameter of 0.05, while sensitivity analyses tested parameters of 0.01, 0.1, and 0.5. Multivariable models for RFS and OS included the dissemination axis (high/intermediate vs. low), AFP ≥ 400 ng/mL, tumor size ≥ 5 cm, and multiple nodules. Portal vein tumor thrombosis (PVTT) was excluded from primary multivariable models due to conceptual and empirical overlap with MVI and near-complete separation. Results of the sensitivity model including PVTT are reported in the Results section. The proportional hazards assumption was verified via scaled Schoenfeld residuals (32).

Time dependence was examined via three approaches. First, Cox proportional hazards models analyzed recurrence within 12 and 24 months of follow-up. Second, a 12-month landmark analysis evaluated recurrence between 12 and 24 months in patients recurrence-free at 12 months. Third, a 24-month landmark analysis assessed recurrence after 24 months of follow-up. Analogous 12-month and 24-month landmark analyses were also performed for OS.

Competing risks were assessed with Aalen-Johansen cumulative incidence functions (33). Fine-Gray subdistribution hazards were estimated via a weighted partial-likelihood implementation in Python (34, 35), with point estimates validated by an independent BFGS optimizer, and cause-specific Cox hazards were included for comparison. In the cause-specific analysis, follow-up duration was defined as the recurrence-free interval for patients with recurrence, and overall survival time for recurrence-free patients. Recurrence was designated as the event of interest, with a 0.05 ridge penalty applied. A detailed implementation note is available in **Supplementary Methods**.

Mediation analysis was performed as an exploratory log-hazard attenuation analysis using a time-fixed binary mediator. We compared the total effect of the high dissemination axis on OS (estimated by a Cox model adjusted for early recurrence) with the direct effect obtained after adding early recurrence to the model. The proportion mediated was calculated as 1 minus the ratio of the direct-effect log-hazard ratio to the total-effect log-hazard ratio, and its 95% confidence interval was estimated from 1, 000 bootstrap resamples with a fixed random seed (2026). This analysis is exploratory: causal mediation assumptions cannot be fully validated in a retrospective cohort, and time-varying mediation effects were not evaluated. The estimate is therefore descriptive and should not be interpreted as evidence of causal mediation.

In this analysis, surrogate-endpoint evidence was limited to the individual patient level. 24-month RFS status was coded as a binary predictor for OS; time-dependent area under the receiver operating characteristic curve (AUCs) at 36, 48, and 60 months were estimated via inverse probability of censoring weighting (36), and Harrell’s concordance index (C-index) was computed. Since 24-month RFS status can only be confirmed after surgery, this analysis is descriptive rather than a baseline prognostic prediction model, and we do not claim trial-level surrogacy here.

All analyses were run in Python 3.12 using packages lifelines (v0.30.3) and scikit-survival (v0.28.0). All statistical tests are two-sided, with p < 0.05 defined as statistically significant. Analyses of the small late-recurrence subgroup are explicitly exploratory, and no formal sample size calculation was performed for this retrospective exploratory study. The regression model adequacy was assessed via the events-per-variable ratio, detailed in the Results section.

## Results

### Cohort

The cohort included 154 patients (136 men, median age 46 years). Most had unfavourable tumour characteristics: 120 (77.9%) had tumours >=5 cm in diameter, 64 (41.6%) had multiple nodules, 75 (48.7%) were Barcelona Clinic Liver Cancer (BCLC) stage B/C, and 36 (23.4%) had PVTT. MVI was detected in 106 patients, distributed as 48 M0, 73 M1, and 33 M2; 43 patients (27.9%) were CK19 positive. Tumour recurrence occurred in 142 patients (92.2%): 118 early recurrence, 24 late recurrence, and 12 had no recurrence. The median observed OS duration was 25 months (interquartile range [IQR] 11.2-44.0, range 2-75), and 83 patients (53.9%) died; all 154 patients had documented OS status. For the multivariable Cox model with 5 predictors, 142 recurrence events and 83 death events gave events-per-variable ratios of 28.4 (RFS analysis) and 16.6 (OS analysis). The competing-risk models incorporated the same set of 5 predictors, with 142 observed recurrence events (events-per-variable ratio = 28.4), and no competing pre-recurrence deaths. Median RFS was 6 months because recurrence was common and occurred early; recurrence-free patients were censored at their last recurrence assessment. At 12 and 24 months, 60 and 36 patients remained recurrence-free, respectively, and 31 recurrence-free patients had follow-up beyond 24 months. Among the 36 patients without early recurrence, median clinical follow-up was 38 months (IQR 27.5-48.0). Baseline characteristics stratified by DA and host-liver axis are shown in **Table 1**.

**Table 1:** Baseline characteristics and outcomes across the six dissemination-axis strata.

Variable	H-H (n=51)	H-L (n=20)	M-H (n=31)	M-L (n=19)	L-H (n=24)	L-L (n=9)	P
MVI grade: M0	0 (0.0)	0 (0.0)	9 (29.0)	6 (31.6)	24 (100.0)	9 (100.0)	<0.001
MVI grade: M1	28 (54.9)	10 (50.0)	22 (71.0)	13 (68.4)	0 (0.0)	0 (0.0)	<0.001
MVI grade: M2	23 (45.1)	10 (50.0)	0 (0.0)	0 (0.0)	0 (0.0)	0 (0.0)	<0.001
Sex, n (%)	Male 48 (94.1); Female 3 (5.9)	Male 18 (90.0); Female 2 (10.0)	Male 26 (83.9); Female 5 (16.1)	Male 14 (73.7); Female 5 (26.3)	Male 22 (91.7); Female 2 (8.3)	Male 8 (88.9); Female 1 (11.1)	0.259
HBsAg, n (%)	Positive 51 (100.0); Negative 0 (0.0)	Positive 10 (50.0); Negative 10 (50.0)	Positive 31 (100.0); Negative 0 (0.0)	Positive 15 (78.9); Negative 4 (21.1)	Positive 24 (100.0); Negative 0 (0.0)	Positive 6 (66.7); Negative 3 (33.3)	<0.001
HBV-DNA, n (%)	≥500 IU/mL 51 (100.0); <500 IU/mL 0 (0.0)	≥500 IU/mL 0 (0.0); <500 IU/mL 20 (100.0)	≥500 IU/mL 31 (100.0); <500 IU/mL 0 (0.0)	≥500 IU/mL 0 (0.0); <500 IU/mL 19 (100.0)	≥500 IU/mL 24 (100.0); <500 IU/mL 0 (0.0)	≥500 IU/mL 2 (22.2); <500 IU/mL 7 (77.8)	<0.001
Tumour size, n (%)	≥5 cm 48 (94.1); <5 cm 3 (5.9)	≥5 cm 19 (95.0); <5 cm 1 (5.0)	≥5 cm 23 (74.2); <5 cm 8 (25.8)	≥5 cm 12 (63.2); <5 cm 7 (36.8)	≥5 cm 11 (45.8); <5 cm 13 (54.2)	≥5 cm 7 (77.8); <5 cm 2 (22.2)	<0.001
Edmondson grade, n (%)	I-II 21 (41.2); III-IV 30 (58.8)	I-II 15 (75.0); III-IV 5 (25.0)	I-II 20 (64.5); III-IV 11 (35.5)	I-II 13 (68.4); III-IV 6 (31.6)	I-II 16 (66.7); III-IV 8 (33.3)	I-II 7 (77.8); III-IV 2 (22.2)	0.037
Tumour number, n (%)	Single 21 (41.2); Multiple 30 (58.8)	Single 8 (40.0); Multiple 12 (60.0)	Single 21 (67.7); Multiple 10 (32.3)	Single 12 (63.2); Multiple 7 (36.8)	Single 20 (83.3); Multiple 4 (16.7)	Single 8 (88.9); Multiple 1 (11.1)	0.001
Tumour capsule, n (%)	Complete 25 (49.0); Incomplete 26 (51.0)	Complete 7 (35.0); Incomplete 13 (65.0)	Complete 21 (67.7); Incomplete 10 (32.3)	Complete 14 (73.7); Incomplete 5 (26.3)	Complete 24 (100.0); Incomplete 0 (0.0)	Complete 9 (100.0); Incomplete 0 (0.0)	<0.001
PVTT, n (%)	Negative 32 (62.7); Positive 19 (37.3)	Negative 9 (45.0); Positive 11 (55.0)	Negative 27 (87.1); Positive 4 (12.9)	Negative 17 (89.5); Positive 2 (10.5)	Negative 24 (100.0); Positive 0 (0.0)	Negative 9 (100.0); Positive 0 (0.0)	<0.001
BCLC stage, n (%)	0+A 14 (27.5); B+C 37 (72.5)	0+A 6 (30.0); B+C 14 (70.0)	0+A 19 (61.3); B+C 12 (38.7)	0+A 11 (57.9); B+C 8 (42.1)	0+A 20 (83.3); B+C 4 (16.7)	0+A 9 (100.0); B+C 0 (0.0)	<0.001
AFP, n (%)	<400 ng/mL 14 (27.5); ≥400 ng/mL 37 (72.5)	<400 ng/mL 4 (20.0); ≥400 ng/mL 16 (80.0)	<400 ng/mL 13 (41.9); ≥400 ng/mL 18 (58.1)	<400 ng/mL 10 (52.6); ≥400 ng/mL 9 (47.4)	<400 ng/mL 19 (79.2); ≥400 ng/mL 5 (20.8)	<400 ng/mL 5 (55.6); ≥400 ng/mL 4 (44.4)	<0.001
M-CTC, n (%)	Negative 3 (5.9); Positive 48 (94.1)	Negative 1 (5.0); Positive 19 (95.0)	Negative 22 (71.0); Positive 9 (29.0)	Negative 13 (68.4); Positive 6 (31.6)	Negative 24 (100.0); Positive 0 (0.0)	Negative 9 (100.0); Positive 0 (0.0)	<0.001
Total CTC, n (%)	Negative 8 (15.7); Positive 43 (84.3)	Negative 9 (45.0); Positive 11 (55.0)	Negative 15 (48.4); Positive 16 (51.6)	Negative 12 (63.2); Positive 7 (36.8)	Negative 16 (66.7); Positive 8 (33.3)	Negative 7 (77.8); Positive 2 (22.2)	<0.001
Cirrhosis, n (%)	Positive 51 (100.0); Negative 0 (0.0)	Positive 19 (95.0); Negative 1 (5.0)	Positive 31 (100.0); Negative 0 (0.0)	Positive 18 (94.7); Negative 1 (5.3)	Positive 24 (100.0); Negative 0 (0.0)	Positive 6 (66.7); Negative 3 (33.3)	<0.001
CK19, n (%)	Negative 33 (64.7); Positive 18 (35.3)	Negative 13 (65.0); Positive 7 (35.0)	Negative 25 (80.6); Positive 6 (19.4)	Negative 14 (73.7); Positive 5 (26.3)	Negative 19 (79.2); Positive 5 (20.8)	Negative 7 (77.8); Positive 2 (22.2)	0.582
Age, years, median (IQR)	46.0 (36.5-54.0)	46.0 (39.8-55.0)	47.0 (38.0-51.5)	46.0 (43.0-54.5)	43.5 (38.8-47.8)	46.0 (44.0-55.0)	0.663
Total CTC count, median (IQR)	10.0 (6.0-16.5)	6.0 (3.8-11.0)	6.0 (4.0-10.5)	4.0 (2.5-6.5)	4.0 (2.0-8.2)	4.0 (1.0-5.0)	<0.001
Mesenchymal CTC, median (IQR)	2.0 (1.0-4.0)	1.0 (1.0-4.0)	0.0 (0.0-1.0)	0.0 (0.0-1.5)	0.0 (0.0-0.0)	0.0 (0.0-0.0)	<0.001
Mixed CTC, median (IQR)	4.0 (2.0-10.5)	2.0 (1.0-4.2)	3.0 (1.0-5.0)	2.0 (1.0-3.0)	2.0 (0.0-3.0)	1.0 (0.0-3.0)	0.001
Epithelial CTC, median (IQR)	2.0 (1.0-4.5)	2.0 (1.0-4.0)	2.0 (0.0-6.5)	1.0 (0.0-3.0)	1.5 (1.0-3.2)	1.0 (0.0-3.0)	0.461
M + mixed CTC, median (IQR)	7.0 (4.0-14.0)	4.0 (2.0-8.2)	3.0 (1.0-6.0)	3.0 (1.0-3.5)	2.0 (0.0-3.0)	1.0 (0.0-3.0)	<0.001
Recurrence, n (%)	50 (98.0)	19 (95.0)	30 (96.8)	18 (94.7)	19 (79.2)	6 (66.7)	
Early recurrence (≤24 months), n (%)	50 (98.0)	19 (95.0)	19 (61.3)	15 (78.9)	13 (54.2)	2 (22.2)	
Late recurrence (>24 months), n (%)	0 (0.0)	0 (0.0)	11 (35.5)	3 (15.8)	6 (25.0)	4 (44.4)	
Death, n (%)	40 (78.4)	13 (65.0)	13 (41.9)	12 (63.2)	3 (12.5)	2 (22.2)	
Recurrence-free survival, months, median (IQR)	3.0 (3.0-5.0)	3.0 (3.0-7.0)	13.0 (7.0-29.0)	10.0 (5.0-18.0)	21.0 (13.2-26.0)	31.0 (24.0-48.0)	
Overall survival, months, median (IQR)	12.0 (6.5-24.0)	14.0 (7.8-23.2)	35.0 (26.0-51.5)	27.0 (19.0-37.0)	40.0 (31.8-48.5)	47.0 (24.0-51.0)	

Data are presented as n (%) or median (IQR). For binary variables, both categories are presented in a single row, formatted as (Category 1: n [%]; Category 2: n [%]). Stratum labels combine the dissemination axis (DA; preoperative M-CTC phenotype plus postoperative pathological MVI grade) and host-liver axis (H = high, M = intermediate, L = low); for example, H-H denotes high dissemination and high host-liver axis. P values were derived from chi-square, Fisher’s exact, or Kruskal-Wallis tests, as appropriate.

### Recurrence-time phenotypes and overall survival

During the observed follow-up period, the three recurrence-time groups differed markedly. Among 118 early recurrence patients, 80 died (67.8%), with a median OS of 18.5 months (Kaplan-Meier median OS estimate: 20.0 months). By contrast, only 3 of 24 late recurrence patients died (12.5%), and no deaths occurred in the 12-patient no-recurrence group. Median OS was not reached for the latter two groups, whose observed median survival times were 49.0 and 39.5 months, respectively. The Kaplan-Meier OS curves separated clearly (log-rank test, p<0.0001; **Figure 1**).

**Figure 1:**
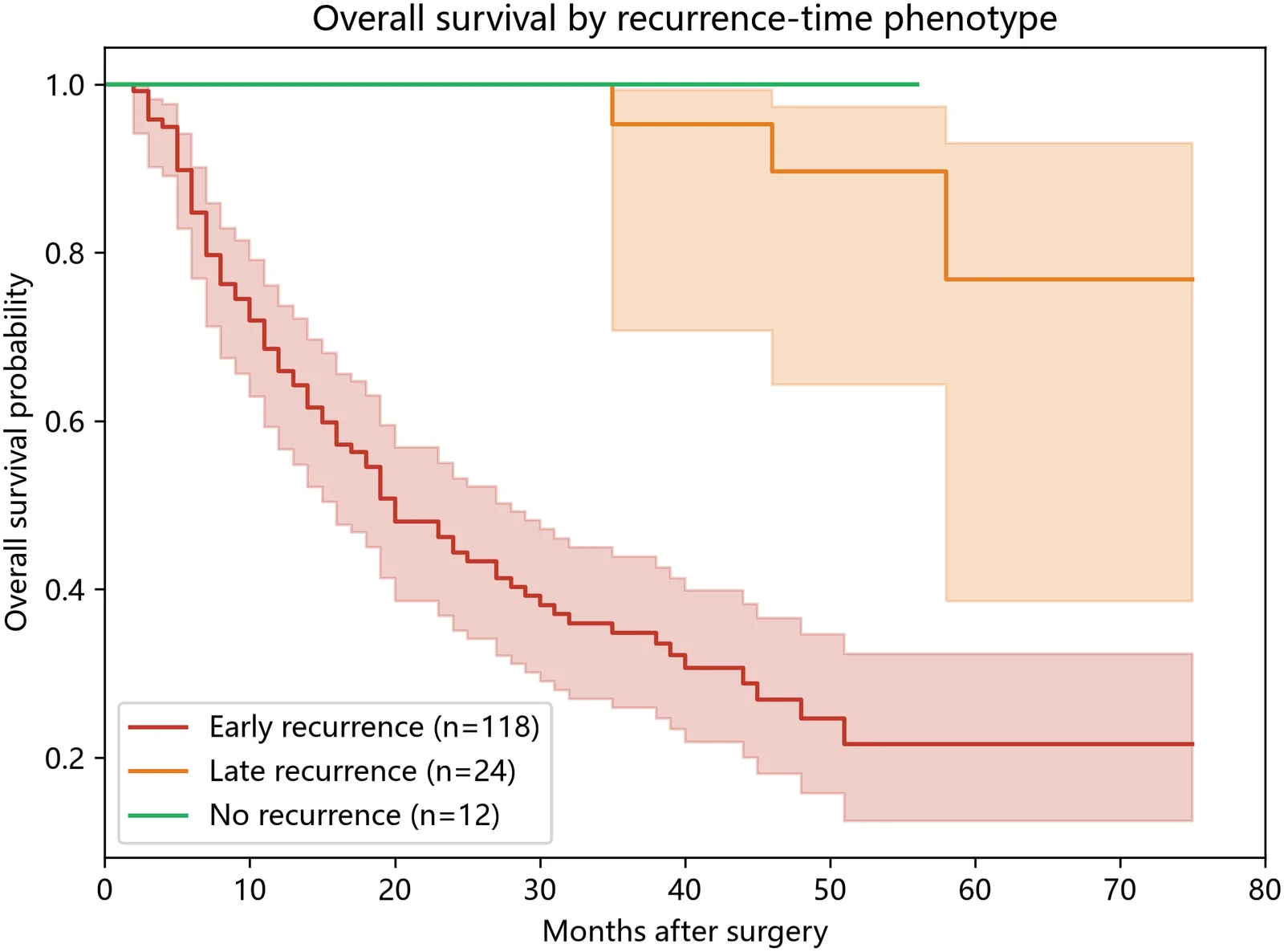
Overall survival according to recurrence-time phenotype (early, late, or no recurrence). Log-rank test p < 0.0001. Numbers at risk are listed in **Supplementary Tables S1-S4**.

In exploratory comparisons, early and late recurrence differed in several tumour features. MVI M2 was detected in 32/118 early recurrences versus 0/24 late recurrences (p=0.0003). Compared with late recurrence, early recurrence had higher frequencies of M-CTC positivity (76/118 vs 4/24; p<0.0001), total CTC count >=6 per 5 mL (79/118 vs 7/24; p=0.001), AFP >=400 ng/mL (79/118 vs 6/24; p=0.0002), BCLC stage B/C (70/118 vs 4/24; p=0.0002), PVTT (35/118 vs 1/24; p=0.009), multiple tumour nodules (60/118 vs 3/24; p=0.0005), incomplete tumour capsule (51/118 vs 2/24; p=0.001), and tumour size >=5 cm (100/118 vs 12/24; p=0.0005). No clear between-group differences were found for age (p=0.118), CK19 expression (p=0.34), cirrhosis (p=0.13), or HBV-DNA status (p=0.46). Among CTC subtypes, EMT-related measures showed the clearest pattern: median counts for early versus late recurrence were 1 vs 0 for M-CTC (p=0.0001), 3 vs 1 for mixed CTC (p=0.006), 5 vs 1.5 for combined M-CTC plus mixed CTC (p=0.0008), and 7 vs 4 for total CTC (p=0.006), with no clear difference in epithelial CTC count (p=0.43). After Benjamini-Hochberg multiple testing adjustment for 19 early-late recurrence comparisons, 13 comparisons retained statistical significance (q<0.05). Given the small late-recurrence subgroup (n=24), these between-group comparisons are exploratory. Although 13 of 19 comparisons remained significant after Benjamini-Hochberg adjustment, the results should not be regarded as confirmatory.

### Dissemination axis and risk stratification

The DA comprised three levels: low, intermediate, and high (**Figure 2**). Across the six dissemination x host-liver strata (**Figure 3**), the 24-month recurrence rate increased from 22.2% in the lowest-risk stratum (low DA, low host-liver axis) to 95-98% in the two highest-risk strata (both with high DA), while median OS declined from 47 months to 12 months. Both RFS and OS differed significantly across the six strata (log-rank test, both p < 0.0001). Overall survival by stratum is shown in **Supplementary Figure S2**.

**Figure 2:**
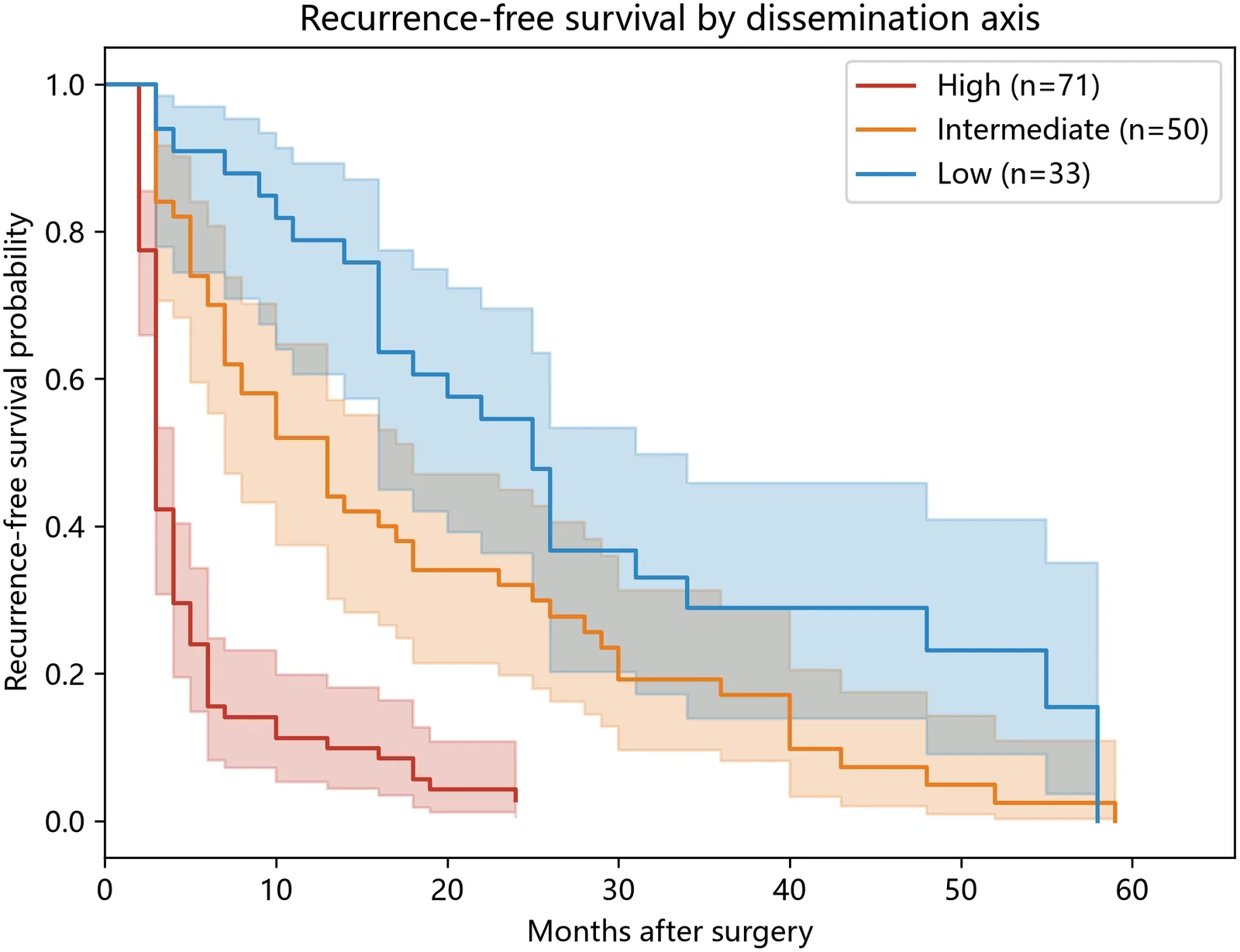
Recurrence-free survival according to DA level (low, intermediate, or high). Log-rank test p < 0.0001. Numbers at risk are listed in **Supplementary Tables S1-S4**.

**Figure 3:**
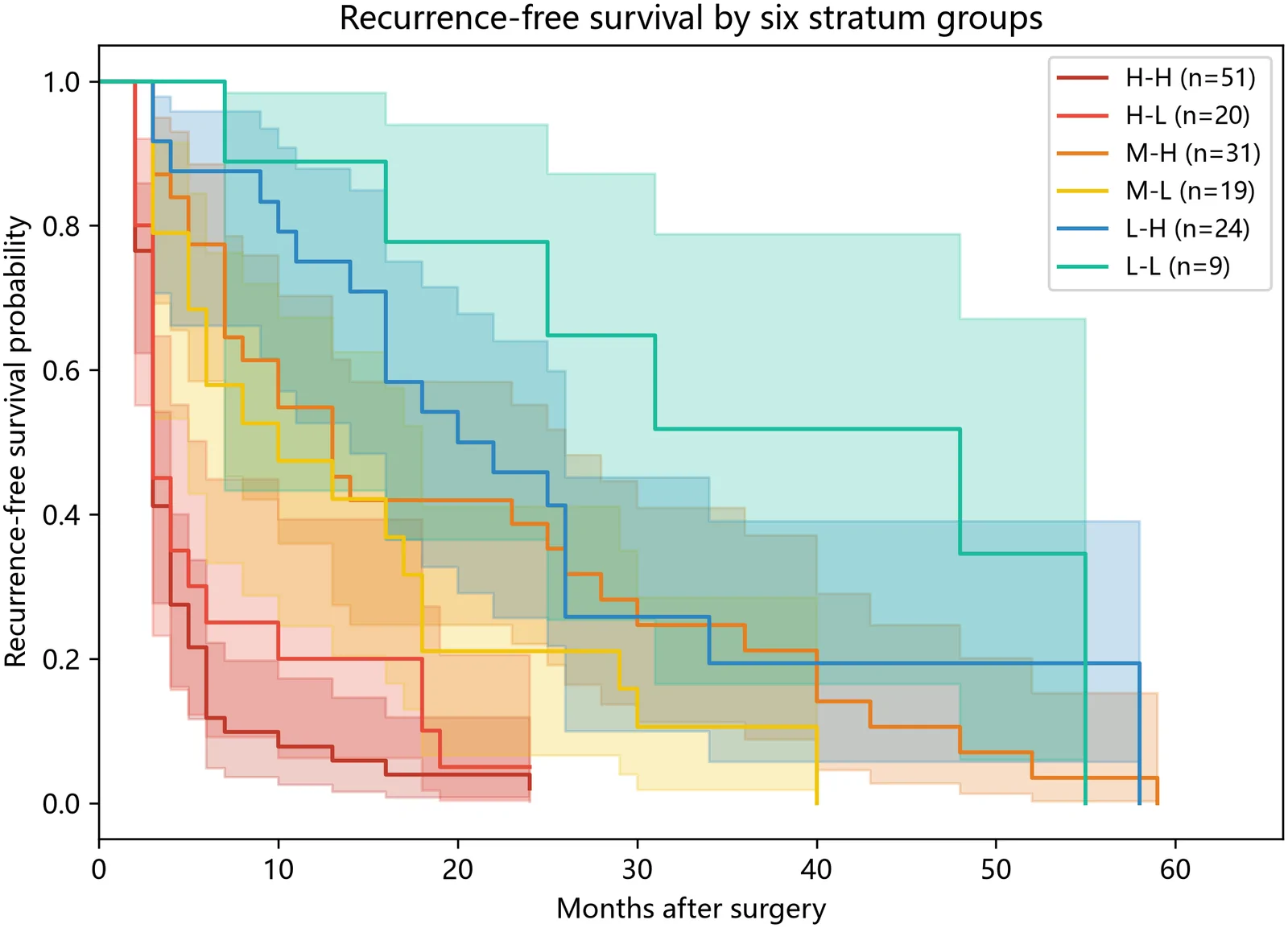
Recurrence-free survival across the six dissemination x host-liver strata. Log-rank test p < 0.0001. Numbers at risk are listed in **Supplementary Tables S1-S4**.

In multivariable Cox regression adjusted for AFP >= 400 ng/mL, tumour diameter >= 5 cm, and multiple tumour nodules, high DA status was identified as an independent predictor of both recurrence-free survival (RFS, HR = 3.72, 95% CI 2.28-6.07; p < 0.0001) and overall survival (OS, HR = 3.25, 95% CI 1.69-6.25; p = 0.0004) (**Table 2**). Intermediate DA status was not an independent significant predictor for either RFS (HR = 1.36, 95% CI 0.87-2.13) or OS (HR = 1.79, 95% CI 0.92-3.48).

**Table 2:** Univariable and multivariable Cox proportional hazards models for recurrence-free survival and overall survival.

Variable	RFS univariable HR (95% CI)	RFS multivariable HR (95% CI)	OS univariable HR (95% CI)	OS multivariable HR (95% CI)
DA high vs low	5.25 (3.34-8.26), p<0.001	3.72 (2.28-6.07), p<0.001	5.46 (3.00-9.97), p<0.001	3.25 (1.69-6.25), p<0.001
DA intermediate vs low	1.50 (0.97-2.33), p=0.069	1.36 (0.87-2.13), p=0.180	2.00 (1.05-3.80), p=0.034	1.79 (0.92-3.48), p=0.084
AFP ≥400 ng/mL	2.22 (1.58-3.12), p<0.001	1.49 (1.04-2.14), p=0.030	2.25 (1.44-3.49), p<0.001	1.51 (0.96-2.39), p=0.076
Tumour size ≥5 cm	2.03 (1.36-3.03), p=0.001	1.57 (1.04-2.38), p=0.031	5.91 (2.84-12.32), p<0.001	4.84 (2.27-10.32), p<0.001
Multiple nodules	2.28 (1.63-3.20), p<0.001	1.60 (1.12-2.28), p=0.009	2.65 (1.74-4.04), p<0.001	1.83 (1.17-2.85), p=0.008

Ridge-penalized Cox models (penalty = 0.05). Multivariable models were adjusted for DA (preoperative M-CTC phenotype plus postoperative pathological MVI grade), AFP >=400 ng/mL, tumour size >=5 cm, and multiple nodules. HR, hazard ratio; CI, confidence interval.

Estimates stayed stable across ridge penalty values from 0.01 to 0.1: for high DA status, hazard ratios for RFS ranged 4.32-3.25, and for OS ranged 4.65-2.65. In sensitivity analysis additionally adjusted for PVTT, high DA status remained significantly associated with RFS (HR = 3.31, 95% CI 1.99-5.50) and OS (HR = 2.68, 95% CI 1.35-5.32), while PVTT produced wide confidence intervals.

### Time windows and landmark analyses

The predictive value of high DA status was time-limited, with effects concentrated in the first 24 months after surgery (**Figure 4**). Its HR for recurrence was 4.74 (95% CI 2.91-7.71) within 12 months, and 4.41 (95% CI 2.85-6.82) within 24 months. Among 60 recurrence-free patients at 12 months, the HR decreased to 2.21 (95% CI 0.91-5.37; p=0.080) between 12 and 24 months after baseline. After 24 months, no valid HR estimate could be generated: all 31 recurrence-free patients followed up after 24 months were not classified in the high-DA group. Scaled Schoenfeld residuals confirmed no violation of the proportional hazards assumption (all p>0.05).

**Figure 4:**
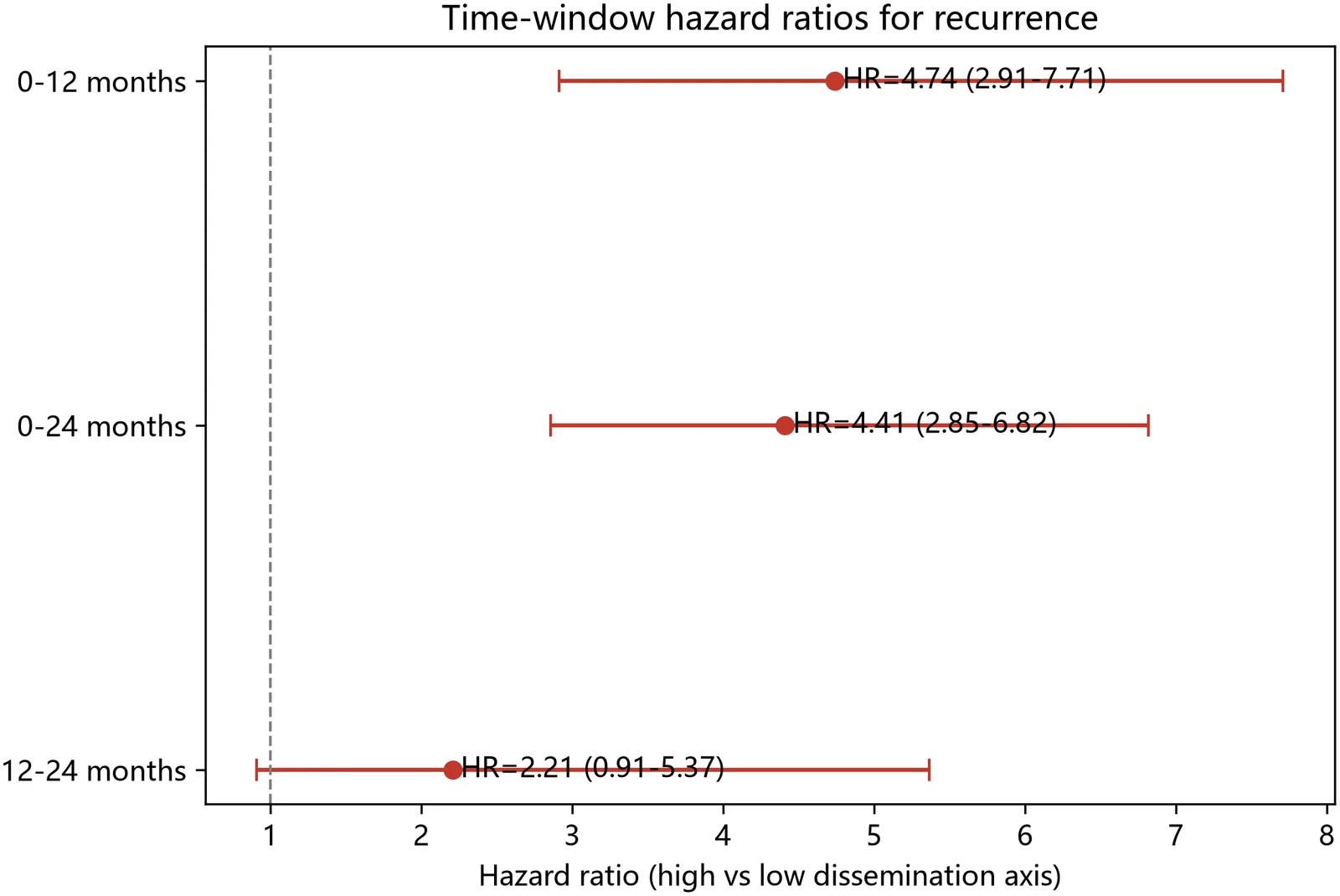
Time-window hazard ratios for recurrence with high DA status (0-12, 0-24, and 12–24 months). No HR could be estimated beyond 24 months, as no patient in this group remained recurrence-free past 24 months.

Overall survival landmark analyses showed consistent results: high DA status significantly predicted mortality in the 12-month analysis (HR 2.42, 95% CI 1.39-4.21; p=0.0017), while no statistically significant association was found in the 24-month analysis (HR 1.43, 95% CI 0.57-3.55; p=0.44).

### Sensitivity analysis at 36 months

In prespecified sensitivity analysis, shifting the late recurrence threshold to 36 months did not change the study conclusions. Early recurrence (within 36 months) occurred in 132 patients (85.7%), with 81 deaths; 10 patients had late recurrence, including 2 deaths; 12 patients remained recurrence-free during follow-up. The observed median survival times for the three groups were 21.5, 53.5, and 39.5 months, respectively. The DA remained a significant predictor of recurrence within 36 months (p<0.001), and no high-DA patients were recurrence-free in the 36-month landmark analysis. Because the 36-month late-recurrence subgroup included only 10 patients, these sensitivity-analysis results are exploratory and should not be considered confirmatory.

### Competing risk

Throughout the entire observed follow-up period, no deaths occurred in the absence of prior tumor recurrence (0/154). Thus, the cumulative incidence of recurrence equals 1 minus the Kaplan-Meier estimate of recurrence-free survival. At 12, 24, and 36 months, cumulative recurrence incidence was 0.887, 0.972, 0.972 for the high-risk group; 0.480, 0.680, 0.829 for the intermediate-risk group; and 0.212, 0.455, 0.711 for the low-risk group (**Figure 5**). For the high-risk group, the univariable and multivariable Fine-Gray subdistribution hazard ratios were 5.84 (95% CI 3.51-9.72) and 4.05 (95% CI 2.34-7.02); the corresponding univariable and multivariable cause-specific hazard ratios were 5.21 (95% CI 3.32-8.19) and 3.68 (95% CI 2.26-6.01), respectively (**Table 3**).

**Figure 5:**
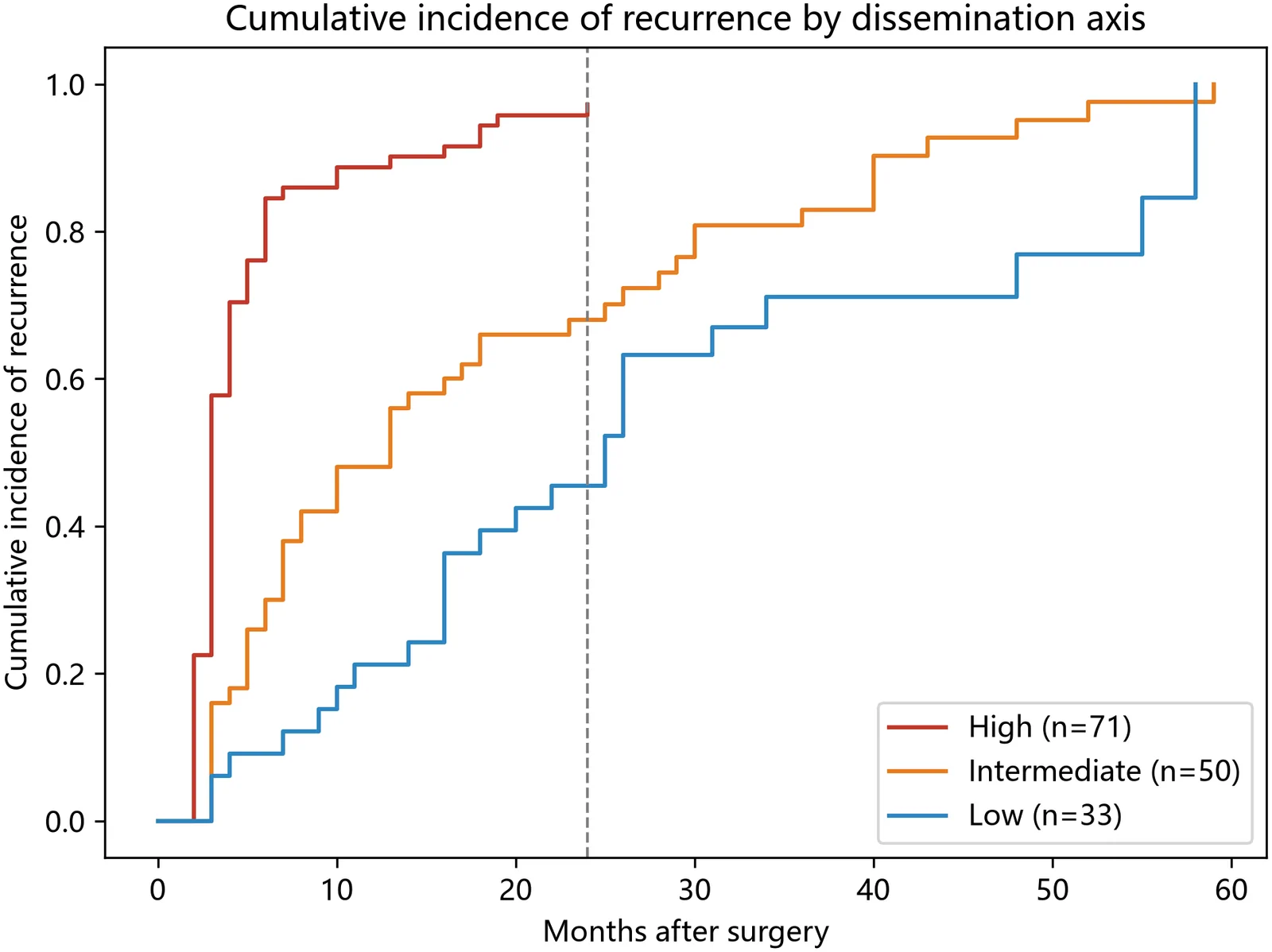
Aalen-Johansen cumulative incidence of recurrence (with death as a competing event), by DA level. As no death preceded recurrence, the curves match the Kaplan-Meier estimates.

**Table 3:** Competing-risk analysis of recurrence.

Panel A: Aalen–Johansen cumulative incidence of recurrence					
Dissemination axis	n	CIF at 12 months	CIF at 24 months		CIF at 36 months
High	71	0.887	0.972		0.972
Intermediate	50	0.480	0.680		0.829
Low	33	0.212	0.455		0.711

Panels B and C: Fine–Gray and cause-specific hazard ratios					
Model	Dissemination axis	Univariable HR (95% CI)		Multivariable HR (95% CI)	
Fine-Gray	High vs low	5.84 (3.51-9.72)		4.05 (2.34-7.02)	
Fine-Gray	Intermediate vs low	1.74 (1.07-2.83)		1.54 (0.93-2.53)	
Cause-specific	High vs low	5.21 (3.32-8.19)		3.68 (2.26-6.01)	

CIF, cumulative incidence function; HR, hazard ratio; CI, confidence interval. Hazard ratios compare high and intermediate DA with low DA; the complete DA can only be calculated after postoperative pathological assessment. The multivariable competing-risk models included 5 predictors and 142 recurrence events (events-per-variable ratio = 28.4); no competing pre-recurrence deaths were recorded.

### Mediation

High DA status yielded an OS HR of 5.46. After adjusting for early recurrence, the direct HR dropped to 3.43, showing that 27.5% of the log-hazard effect was attenuated by early recurrence, consistent with bootstrap estimation (median 27.6%, 95% CI 15.5%-44.9%). Among 83 total deaths, 80 (96.4%) occurred in patients with early recurrence. The estimate stayed stable across all tested ridge penalties (0.197-0.277) and was higher when 12-month recurrence status was specified as the mediator (0.369 at the primary penalty). This attenuation analysis is exploratory and should not be interpreted as evidence of causal mediation.

### Surrogate endpoint

The 24-month RFS signature was used to stratify the OS cohort (**Figure 6**). The time-dependent AUC values at 36, 48, and 60 months were 0.732, 0.807, and 0.862 respectively, with a simple arithmetic mean of 0.800. The time-averaged AUC calculated across the censoring distribution was 0.755, and the Harrell’s C-index was 0.657. This finding provides patient-level evidence that 24-month RFS is a candidate early endpoint for perioperative clinical trials, rather than conclusive proof of trial-level surrogacy. Restricting the analysis to patients with at least 24 months of follow-up or patients with early recurrence did not meaningfully change effect estimates.

**Figure 6:**
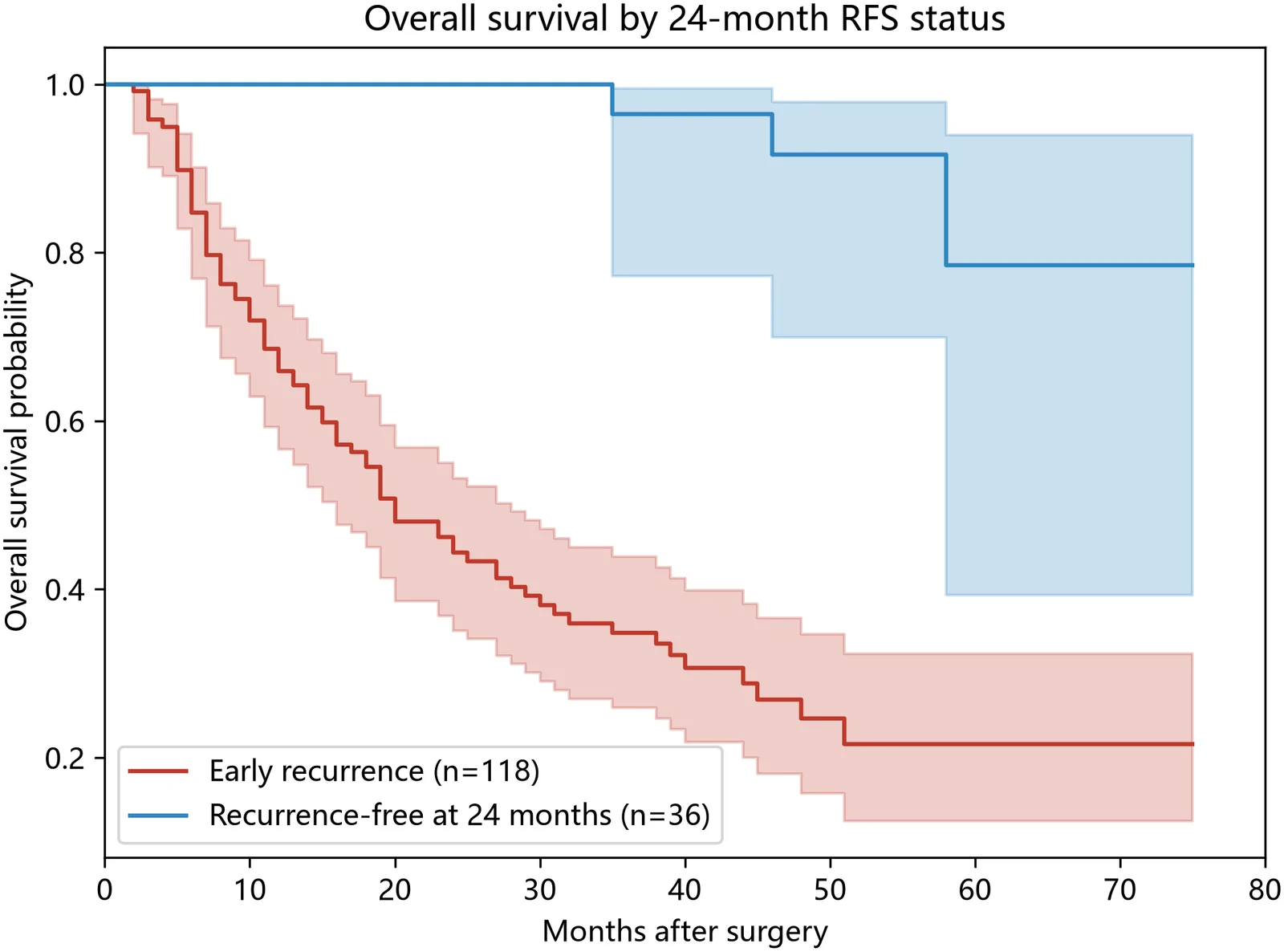
Overall survival according to 24-month recurrence-free survival status-a patient-level surrogate endpoint. Numbers at risk are listed in **Supplementary Tables S1-S4**.

## Discussion

This study reports an observational finding in patients undergoing curative resection for HCC: recurrence within 24 months was associated with poorer overall survival than recurrence later or no recurrence. A DA integrating a preoperative CTC phenotype with postoperative pathological MVI grade stratified patients into groups with markedly different early-recurrence risk, and this association was concentrated in the first two postoperative years. Unlike longitudinal postoperative CTC monitoring (22) or composite blood-based recurrence risk scores (23), this integrated measure can be calculated after pathology review without serial postoperative sampling. The association was examined using mediation, competing-risk, and patient-level endpoint analyses; these analyses were complementary but exploratory. Because all patients with high DA status had already recurred by 24 months, no further first-recurrence events could be observed in this group; recurrence continued to accrue in lower-risk groups. During the observed follow-up period, no deaths occurred without prior recurrence, a pattern consistent with early recurrence accounting for part of the association between the DA and mortality. However, mediation used a time-fixed mediator and cannot establish causality. Because this study lacked external validation, the findings remain hypothesis-generating and require independent validation before clinical use.

Why might recurrence timing be prognostically important? Early recurrence may reflect occult vascular dissemination present at the time of surgery (5, 6, 37). In exploratory comparisons, MVI M2 and M-CTC positivity were more common in patients with early recurrence, and neither marker retained a clear association beyond 24 months after resection. These patterns are consistent with shared underlying biology. Our mediation analysis adopts a time-fixed mediator and log-hazard attenuation approach, and remains descriptive, incapable of validating causal mediation. The residual, unattenuated association may reflect unmeasured tumour burden, host factors, or intrinsic biological aggressiveness.

This study extends the current landscape of CTC-based recurrence prediction. Prior studies have primarily addressed two questions: “Will recurrence occur?” and “When is it likely to occur?” For instance, longitudinal CTC monitoring has demonstrated utility in detecting minimal residual disease and predicting early recurrence following curative resection (22), while composite blood-based scores including the CTC-ALRI have shown prognostic value for recurrence risk (23). Building on this foundation, our study advances a third question: does the timing of recurrence, not merely its occurrence, discriminate between lethal and non-lethal disease trajectories? We show that a DA integrating EMT-associated CTC burden and MVI grade identifies patients at risk for this high-risk temporal pattern. The two paradigms are complementary: postoperative dynamic monitoring refines risk assessment over time, whereas our integrated DA provides risk stratification soon after surgery, once pathology is available. Given the heterogeneity in defining early recurrence across studies (e.g., 12-month thresholds in some reports (27)), we prespecified 24 months as the primary cutoff and confirmed its robustness through a 36-month sensitivity analysis.

The host-liver axis requires rigorous evaluation. Cirrhosis and elevated baseline HBV-DNA load were common in the cohort but could not distinguish early (<=24 months) from late (>24 months) recurrence. This null finding reflects the likely biological role of this axis: it captures liver disease-driven background risk, not tumour-intrinsic aggressiveness. Thus, these variables were excluded from the predictive model. Clinically, all patients after curative resection need long-term surveillance regardless of DA risk. Supporting this, 24 of 31 patients recurrence-free at 24 months and followed beyond that point later recurred.

Several methodological points merit brief clarification. First, no deaths occurred before recurrence, so cumulative incidence of recurrence equaled 1 − Kaplan–Meier RFS. Fine–Gray and cause-specific models were included for completeness and showed consistent direction. Second, mediation used log-hazard attenuation—a descriptive metric—with a bootstrap 95% CI (15.5%–44.9%), indicating uncertainty; causal claims are limited by untestable assumptions in this retrospective study. Third, surrogate analysis was restricted to the patient level. Our data suggest 24-month RFS as a candidate surrogate for OS in perioperative HCC trials—but validation requires prospective trial evidence, not observational data.

The key limitations of this work include its single-centre, retrospective design with a relatively small sample size (n = 154) and the absence of external validation. Furthermore, the high proportion of unfavourable tumour characteristics in the cohort may restrict the generalizability of these results to populations consisting predominantly of patients with early-stage HCC (77.9% tumours ≥5 cm, 48.7% BCLC stage B/C, and 23.4% PVTT). Other limitations include (1): finite follow-up, such that some late recurrences that may become lethal occur beyond the observation period, so mortality differences reflect the study’s time horizon (2); all analyses are exploratory, with confidence intervals reported to quantify uncertainty (3); no *a priori* sample-size calculation, but events-per-variable ratios were 28.4 (RFS) and 16.6 (OS), both >10 (4); no multiplicity correction in primary analyses, although key findings remained significant after FDR adjustment (5); missing or unavailable information includes recurrence site, serial postoperative CTCs, ALRI, cause-of-death adjudication, exact loss-to-follow-up status, and baseline characteristics of excluded patients; and (6) Fine-Gray estimates were validated with dual optimizers. The composite DA is exploratory because the component thresholds were pre-specified, but the three-level combination rule was not a formally registered protocol endpoint and has not undergone external validation; it requires independent external validation before clinical use. Early-versus-late recurrence comparisons, particularly in the 36-month sensitivity analysis, were based on small sample sizes and wide confidence intervals and should therefore be regarded as exploratory. Since the duration of follow-up was finite, the occurrence of subsequent deaths among patients who remained recurrence-free by the end of the observation period cannot be ruled out. The causes of death were not adjudicated, and administrative censoring events cannot be differentiated from loss to follow-up in the de-identified dataset. If censoring were informative, RFS estimates could be biased; this assumption cannot be fully verified in a retrospective cohort.

Despite these limitations, our findings support a preliminary, biologically grounded surveillance framework for hypothesis testing. For patients with high DA status, quarterly imaging during year 1, extended through year 2, may be considered, alongside referral to adjuvant therapy trials in eligible candidates. For those with low DA status, less intensive initial imaging may be reasonable, but long-term surveillance remains essential: late recurrence occurred in 15.8% (24/152) of patients, including patients with favourable baseline tumour biology (e.g., low CK19 expression and absence of microvascular invasion). The dual-track strategy is detailed in **Supplementary Figure S3**. All recommendations are hypothesis-generating and await prospective validation.

## Conclusion

In this exploratory, single-centre, retrospective cohort of 154 patients with resected HCC, recurrence timing distinguished lethal from non-lethal trajectories during observed follow-up. No deaths occurred without prior recurrence (0/154). The DA, combining a preoperative CTC phenotype with postoperative MVI grade, identified a high-risk early-recurrence group. Its association with survival was partly attenuated by early recurrence in an exploratory analysis and should not be interpreted as evidence of causal mediation. These hypothesis-generating findings support evaluating risk-stratified surveillance and 24-month RFS as an early endpoint in perioperative trials, but external validation is required before clinical use.

Supplementary Methods: Fine-Gray subdistribution hazard estimation—implemented via weighted partial likelihood, with censoring weights applied; model convergence confirmed using BFGS optimization, and results cross-validated with R’s cmprsk package.

## Data Availability

The datasets presented in this study are not publicly available because they contain patient-level clinical information subject to privacy and ethical restrictions. De-identified data may be made available from the corresponding author upon reasonable request and subject to the relevant institutional ethics approval and data-use agreement. Requests to access the datasets should be directed to Bangde Xiang (xiangbangde@gxmu.edu.cn).

## Supplementary material

Supplementary Figure 1 Participant flow diagram.

Supplementary Figure 2 Overall survival across the six dissemination × host-liver strata. Numbers at risk are listed in **Supplementary Tables S1**–**S4**.

Supplementary Figure 3 Dual-track surveillance strategy matrix.
