# Supplementary material for: All deaths follow recurrence, but not all recurrences are fatal: a retrospective cohort study of recurrence timing and survival after curative resection of hepatocellular carcinoma
Source: Front Oncol. 2026 Oct 28;16:1991522. doi: 10.3389/fonc.2026.1991522 (PMC13751409; doi:10.3389/fonc.2026.1991522)
Supplement: Supplementary file 1 [file SupplementaryFile1.docx]

# Supplementary Methods: Fine-Gray subdistribution hazard estimation

**These Supplementary Methods accompany the manuscript “All deaths follow recurrence, but not all recurrences are fatal: a retrospective cohort study of recurrence timing and survival after curative resection of hepatocellular carcinoma” (Frontiers in Oncology).**

## Purpose

Fine-Gray subdistribution hazards were estimated to describe the effect of the dissemination axis on the cumulative incidence of recurrence when death without prior recurrence is treated as a competing event.

## Event definitions

- Event 1 (of interest): recurrence.
- Event 2 (competing): death without prior recurrence.
- Censored: no event at last follow-up.

In the present cohort, no death without prior recurrence was observed (0/154). All 83 deaths occurred after recurrence. Cumulative incidence of recurrence was therefore identical to the Kaplan-Meier estimate, and the Fine-Gray results are reported for methodological completeness.

## Censoring weights

Let G(t) = P(C > t) be the survival function of the censoring distribution, estimated by the Kaplan-Meier method with censoring (no event) as the outcome. In the standard formulation, a subject who experiences the event of interest receives weight 1 at its event time, and subjects who experience the competing event or are censored contribute with weight G(t)/G(min(t, T_j)). Because no competing event was observed in the present cohort, these weights reduce to 1 in all risk sets, and the weighted partial likelihood is equivalent to a ridge-penalised Cox partial likelihood with Breslow-type handling of tied event times. Cumulative incidence of recurrence therefore equalled the Kaplan-Meier estimate, and the Fine-Gray estimates are reported for methodological completeness.

## Model

The subdistribution hazard is modeled as

lambda_1(t | X) = lambda_10(t) * exp(X beta)

and beta is estimated by maximizing the weighted partial likelihood

LL(beta) = sum_k [ sum_{i in D_k} X_i beta - n_k log( sum_{j in R_k} w_jk exp(X_j beta) ) ]

where D_k is the set of subjects with event 1 at time t_k, R_k is the risk set (subjects with T_j >= t_k), and n_k is the number of events at t_k. A ridge penalty of 0.05 was applied to stabilize estimation (0.5 × λ × β^T β was subtracted from the log-likelihood and λ was added to the diagonal of the Hessian). Parameters were obtained by Newton-Raphson iteration; standard errors came from the inverse of the penalized observed information matrix.

## Independent confirmation

Point estimates were confirmed with an independent BFGS optimizer (scipy.optimize.minimize) using the same weighted partial likelihood. Point estimates agreed exactly (univariable high-axis HR 5.84, intermediate 1.74; multivariable high-axis HR 4.05). Standard errors were taken from the analytic Newton-Raphson information matrix because the BFGS inverse-Hessian approximation was not reliable for this penalized likelihood.

## Software

Python 3.12 with numpy 2.3.5, pandas 2.3.3, lifelines 0.30.3 (Kaplan-Meier censoring distribution), and scipy 1.18.0 (BFGS). An independent R cmprsk cross-check was performed to confirm the point estimates.
